# A Practical Primer: Raman Spectroscopy for Monitoring of Photopolymerization Systems

**DOI:** 10.3390/polym15183835

**Published:** 2023-09-20

**Authors:** Julie L. P. Jessop

**Affiliations:** Swalm School of Chemical Engineering, Mississippi State University, Starkville, MS 39762, USA; jessop@che.msstate.edu; Tel.: +1-662-325-2480

**Keywords:** Raman confocal microscopy, free-radical photopolymerization, cationic photopolymerization

## Abstract

Photopolymerization systems provide compelling advantages for industrial applications due to their fast reaction kinetics, wide selection of monomers for physical property development, and energy-efficient initiation via illumination. These same advantages can present challenges when attempting to monitor these reactions or characterize their resulting polymers; however, Raman spectroscopy can provide the flexibility and resolution needed. In this overview, Raman spectroscopy is compared to common characterization techniques, such as photo-differential scanning calorimetry and infrared spectroscopy, highlighting advantages of Raman spectroscopy. Examples are provided of how Raman spectroscopy has been used to monitor photopolymerizations and to provide insight on the impact of monomer chemistry and processing conditions, as well as paired with other techniques to elucidate physical properties. Finally, practical tips are provided for applying Raman spectroscopy and microscopy in photopolymerization systems.

## 1. Introduction

Photopolymerization systems provide compelling advantages for industrial applications due to their fast reaction kinetics, wide selection of monomers for physical property development, and energy-efficient initiation via illumination [1,2]. These same advantages can present challenges when attempting to monitor these reactions or characterize their resulting polymers; however, Raman spectroscopy can provide the flexibility and resolution needed. In particular, the commercial availability of fiber-optic-based Raman systems has opened the door for real-time and in situ monitoring of photopolymerizations for industrial and academic scientists and engineers alike.

Raman spectroscopy is based upon the rotational and vibrational transitions in molecules and is particularly well suited for the detection of chemical bond changes during polymerization [3,4,5,6]. The short intrinsic time scale of this method enables the monitoring of rapid reactions in real time. In addition, minimal sample preparation is required, and a variety of sample geometries may be analyzed (such as thin films or thick samples), as Raman spectroscopy is based on light scattering principles rather than absorption or transmission. Finally, improved instrumentation allows for enhanced signal collection. Modern detectors are significantly more sensitive, and lasers enable the isolation of one wavelength, which produces a clearer, stronger Raman spectrum.

Raman spectroscopy has proven valuable in polymer identification, reaction monitoring, and studies of polymer composites, blends, coatings, and orientated films [4,7]. However, with photopolymerization systems, the need for an illumination source to initiate the chain reaction adds complexity when attempting to apply an analytical technique to follow the reaction in real time. Adaptions to heat-based analytical techniques, such as differential scanning calorimetry [8], optical pyrometry [9], and thin-film calorimetry [10], are straightforward. In these techniques, an illumination source is mounted above the sample, and the heat flow from the exothermic reaction is measured and directly related to the rate of polymerization. Adaptations to spectroscopy-based analytical techniques, such as infrared [11], fluorescence [12], and Raman spectroscopy [6], require more finesse. In these techniques, the illumination source must be placed in such a way as to minimize or prevent its photons from impinging upon the sensitive detectors (e.g., perpendicular to the beam used to generate the spectroscopic signal). The resulting spectra must be analyzed to draw a direct relationship between spectral feature changes due to the reaction and the conversion from monomer to polymer.

Although the heat-based analytical techniques are relatively easy and inexpensive, spectroscopy-based techniques can provide much more information and resolution. For example, Raman spectroscopy can be used to calculate the conversion of the methacrylate (via free-radical photopolymerization) and of the epoxide (via cationic photopolymerization) during a hybrid photopolymerization since the peaks associated with each reactive group can be separately processed [13]. With photo-differential scanning calorimetry (PDSC), the exotherms from the simultaneous reactions overlap, making it impossible to determine the individual conversion of the two monomer types. Moreover, even in homopolymerization systems, the conversion results from PDSC measurements can be uncertain due to assumptions regarding the average monomer functionality and a standard heat of polymerization, ΔH_p_, for the general type of functional group present. For example, an absolute conversion cannot be calculated from a PDSC trace of the commercial monomer PETA-K, which contains an unspecified mixture of tri- and tetra-acrylate esters of pentaerythritol (i.e., the ΔH_p_ for PETA-K is somewhere between 2.33 and 3.11 × 10^5^ J/mol if ΔH_p_ = 7.78 × 10^4^ J/mol is used for a generic acrylate vinyl bond). Conversely, conversion calculations from Raman spectra do not require prior knowledge of the composition for the multi-functional acrylate mixture. In addition, tracking dark cure (i.e., continued reaction after shuttering of the light) in epoxide systems would be impossible over long timeframes for PDSC. It is not feasible to keep the sample in the PDSC for days, months, and/or years. However, with Raman spectroscopy, it is simply a matter of storing the samples and taking them out occasionally to measure conversion changes over time [14]. 

Vibrational spectroscopies, such as infrared (IR) and Raman spectroscopy, are especially useful analytical techniques for photopolymerization systems in that both qualitative (i.e., what is present?) and quantitative (i.e., how much is present?) information is provided. The two techniques rely on different mechanisms: Raman is a scattering technique based on the polarizability of molecular bonds, and IR is an absorption technique based on the dipole moment of molecular bonds [3]. Thus, the two are considered complementary techniques in that some spectral information is provided by both, while other information is only discernable through one or the other technique. For example, both techniques can be used to calculate the conversion of methacrylate monomers; however, Raman is a better choice for aqueous systems since water is Raman-inactive but can obscure important spectral details in IR measurements [15]. Because the Raman effect is comparatively small (only one in a billion photons results in the Raman scattering effect), it has often been overlooked as a practical analytical technique. However, the availability of more sensitive detectors to capture the small number of scattered photons and the wider selection of lasers to avoid inducing fluorescence, which can overwhelm the Raman signal, enable Raman spectroscopy to compete with IR spectroscopy more effectively. For example, both types of spectroscopy are established techniques in dental research that focuses on photopolymerized resins and composites [16]. In general, Raman spectroscopy provides more flexibility for sample presentation than IR spectroscopy, in which sandwiching between salt plates or a thin film on an attenuated total reflectance (ATR) crystal may be required. Additionally, Raman microscopy provides better spatial resolution and depth penetration than IR microscopy [17].

This article is written for the scientist or engineer who wants to use Raman spectroscopy to explore photopolymerization systems. It is not meant to be an exhaustive review; the references cited are intended to provide examples of how Raman spectroscopy has been applied in photopolymerization systems, as well as starting points for a more thorough foray depending on the needs of the reader. The information contained in this article leverages the author’s extensive use of Raman spectroscopy and microscopy over the past decades and includes practical considerations and tips on how to implement the technique for the unique challenges presented by photopolymerization systems.

## 2. Raman Measurements in Photopolymerization Systems

### 2.1. Conversion Measurements

Conversion measurements are the most straightforward and common kinetic values obtained through Raman spectroscopy. The calculation requires identification of a reaction (*rxn*) peak, which is directly related to the reactive moiety on the monomer, and a reference (*ref*) peak, which is associated with molecular bonds not affected by the polymerization. Conversion is then calculated from the ratio of the peak intensity (or peak area) of these reaction and reference peaks:

(1) Conversion,α=1−IrxntIreftIrxn0Iref0

where *I*(*t*) denotes the peak intensity at time *t*, and *I*(0) represents the initial peak intensity before photopolymerization begins. This ratio method corrects for density changes and instrumental variations and is necessary when making conversion comparisons at different sampling times (e.g., in photopolymerized-methacrylate-based dental adhesive resins before and after water storage [18]).

Suitable reference and reaction peaks can be identified through Raman reference books [19,20], literature values, and/or experiments. Raman peak selections are presented in Table 1 for common classes of monomers undergoing photopolymerizations. The reaction peak is relatively standard for each type of reactive moiety (e.g., ~1640 cm^−1^ for C=C bonds in (meth)acrylates and ~790 cm^−1^ for cycloaliphatic epoxides); however, the reference peak differs depending on the structure of the monomer. For example, 3-ethyl-3-phenoxymethyl oxetane (POX) includes a phenyl group that provides a strong reference peak at 980 cm^−1^, while 3-ethyl-3-[(2-ethylhexyloxy)methyl] oxetane (EHOX) has aliphatic groups that present a reference peak at 1450 cm^−1^ [21]. Both monomers contain the oxetane moiety, which provides the reaction peak at 1150 cm^−1^. Ideally, the reference and reaction peaks should be close to one another so that they are affected similarly by any spectral perturbations (fluorescence, changes in density and refractive index during polymerization, etc.). Thus, they both are typically selected from the fingerprint region (i.e., 200–1800 cm^−1^). In addition, the reference peak and reaction peaks should be selected from bonds that are present on the same molecule to avoid issues due to concentration gradients or inhomogeneities in the sample. 

**Figure 1:**
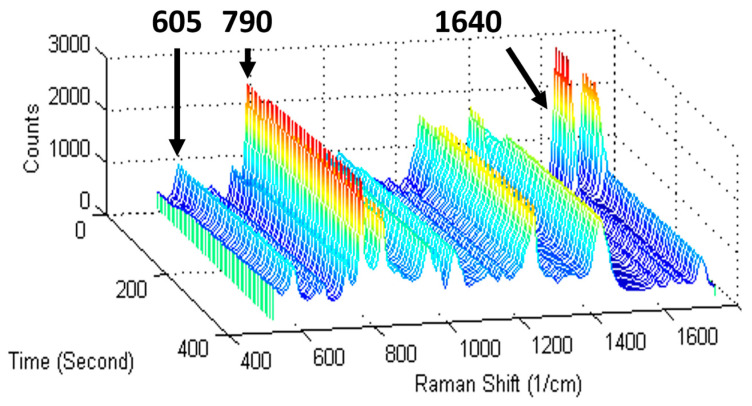
Raman waterfall plots can confirm selection of suitable reaction and reference peaks for conversion calculations. Shown are real-time Raman spectra taken during free-radical photopolymerization of the epoxide–acrylate hybrid monomer 3,4-epoxy-cyclohexyl-methyl methacrylate (METHB) (adapted from Ref. [13]). The reaction peak at 1640 cm^−1^ (methacrylate C=C) decreases with time, while the reference peak at 605 cm^−1^ (C–C–O group) remains constant. Moreover, the reaction peak at 790 cm^−1^ (epoxide ring) does not change, since a cationic photoinitiator was not present in the formulation.

To verify suitability of reaction and reference peak selections, a conversion calibration curve relating the monomer mass fraction to the Raman intensity ratio (in Equation (1)) can be constructed by dissolving various amounts of polymer in its monomer (e.g., PHEMA in HEMA [18]). However, most systems do not have commercially available polymers, and higher mass fractions of polymers take a long time to dissolve in the monomer. Thus, real-time Raman spectroscopy of the photopolymerization provides a more feasible confirmation that the choice of reference and reaction peaks is appropriate. Visual inspection of a 3-D waterfall plot (see Figure 1 for a representative example) is a facile way to verify that the reaction peak decreases with illumination time, while the reference peak remains constant. This confirmation is important since Raman peaks attributed to non-reactive bonds adjacent to the reactive moiety may change during the reaction. For example, the (meth)acrylate carbonyl peak at 1720 cm^−1^ decreases with reaction of the C=C; thus, it is a poor reference peak but can be effectively used as an apparent reaction peak [25]. If conversion measurements are calculated from real-time Raman spectra with a stable baseline, then Equation (1) can be simplified as follows since *I_ref_*(*t*)/*I_ref_*(0) ≈ 1 [26]:

(2) $$Conversion,\alpha =1-\frac{I_{rxn}\left(t\right)}{I_{rxn}\left(0\right)}$$

However, if the baseline changes during the photopolymerization or if conversion measurements are calculated at discrete points in time (e.g., Raman spectra are collected at regular intervals after shuttering the light source to determine dark cure in epoxides [14]), the reference peak values must be included in the conversion equation.

**Figure 2:**
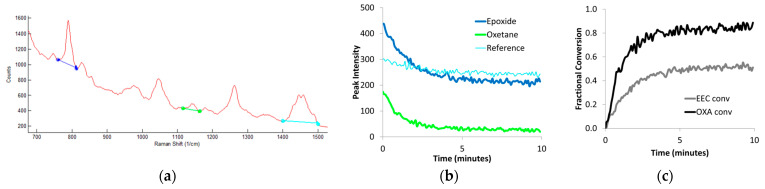
Conversion profiles (**c**) are obtained by measuring peak height or area (**b**) from raw Raman spectra (**a**) and inserting these values into Equation (1). Here, the conversions of the diepoxide EEC and 3-ethyl-3-hydroxymethyl oxetane (OXA) in a comonomer formulation are individually calculated from the same data set using the reaction peak for a cycloaliphatic epoxide (blue line, 790 cm^−1^) and for an oxetane (green line, 1150 cm^−1^) with a common reference peak (turquoise line, 1450 cm^−1^) (adapted from Ref. [24]).

Once Raman spectra have been collected, the data must be transformed to obtain conversion measurements (see Figure 2 for a representative example). Software packages included with the Raman instrument are often helpful with this process. For each spectrum, a spectral range can be selected for each peak, and either the peak intensity or the peak area can be automatically calculated. In some software packages, these peak measurements can be mathematically manipulated to calculate conversion using Equation (1). Otherwise, the peak measurements can be exported to a spreadsheet (e.g., Excel) for the conversion calculation. If the reaction or reference peak is not well resolved or on a similar magnitude as the noise in the spectrum, then a more rigorous data processing method may be required, such as taking the second derivative of the spectrum [27]. For optimal results, the pre-polymerization ratio, *I_rxn_*(0)/*I_ref_*(0), must be reliable; therefore, extra care must be taken in collecting these data. Longer exposure times and multiple accumulations should provide an average spectrum with an appropriately high signal-to-noise ratio (S/N). When processing real-time data, some smoothing of the final profiles may be necessary (e.g., a five-point moving average) since lower concentrations have small peak intensities, which introduce noise in the conversion data [26].

**Figure 3:**
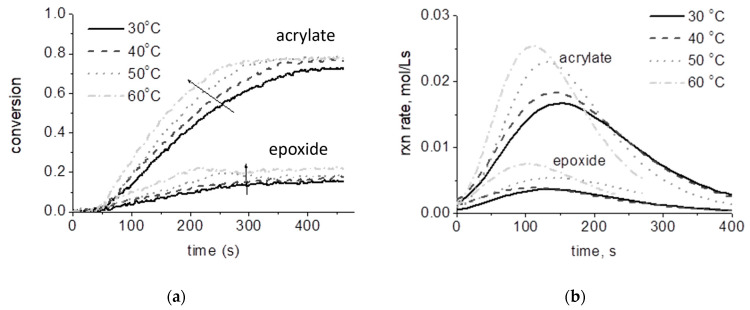
Rate of photopolymerization profiles (**b**) is obtained by differentiating conversion profiles (**a**). Here, the effect of temperature on the hybrid photopolymerization of METHB, which contains both epoxide and methacrylate moieties, is demonstrated (adapted from Ref. [35]).

Conversion data can be used directly to study the effect of formulation choices and processing variables on kinetic outcomes. Because individual reactive moieties can often be resolved, Raman monitoring is useful beyond homopolymerizations for more complicated systems involving copolymerization or hybrid polymerization. For example, improved kinetic outcomes were realized through comonomer systems of EEC and EPOH [23] and of EEC and oxetanes [24]. In addition, the synergy between free-radical and cationic-ring-opening photopolymerizations has been characterized for epoxide–acrylate hybrid monomers [13,28] and systems [26], as well as oxetane–acrylate hybrid systems [29]. Likewise, the impact of other formulation components such as photoinitiators, impurities, and additives can be explored. For example, in cationic photopolymerizations, photoinitiator concentration [13], photoinitiator structure [30], water concentration [28], and alcohol size and functionality [31] play large roles in the extent and rate of polymerization. In free-radical photopolymerizations, examples include demonstrating the effect of crosslinker concentration on conversion for droplet polymerization in a microfluidic device [32] and the impact of TiO_2_ nanoparticle loading on photopolymerization outcomes for acrylate materials intended for micro-optical devices [33]. Processing variables that affect conversion results include reaction temperature (see Figure 3a), spectral output of light source, effective irradiance, illumination time, annealing conditions, etc. For example, a central composite design used conversion as a response to model the effects of effective irradiance, exposure time, and sample depth in shadow cure of EEC [14]. Annealing time and temperature have also been optimized for ECC and another diepoxide based on conversion outcomes [34].

### 2.2. Other Kinetic Data

Conversion profiles can be further manipulated to obtain additional kinetic information. The rate of polymerization (*R_p_*) is calculated from the first derivative of the conversion profile (see Figure 3 for a representative example). Because experimental data are often noisy, the derivative of the best fit trendline is used rather than individual data points. For example, a triple exponential expression was used for DVE-3 [6], while a four-parameter logistic function was used for EEC [31]. From the *R_p_* profiles, the propagation rate constant (*k_p_*) can be calculated by rearranging the propagation mechanistic equation:

(3) $$k_{p}=\frac{R_{p}}{\left[M^{*}\right]\left[M\right]}$$

The monomer concentration, [*M*], is known, and the active center concentration, [M*], can be estimated [6,36]. In addition, the first-order kinetic rate constant for termination/trapping of active centers (*k_t_*_/*t*_) can be found by dividing *R_p_*/[*M*] into its first derivative [36]:

(4) kt/t=dRpMdtRpM

Moreover, real-time Raman conversion data can be used to calculate reactivity ratios (*r_i_*) for photopolymerizations of comonomer formulations [21]. These conversion data enable calculation of instantaneous mole fractions of the monomer in the feed (*f_i_*) and in the copolymer (*F_i_*), from which the reactivity ratios are estimated:

(5) $$F_{1}=1-F_{2}=\frac{r_{1}f_{1}^{2}-f_{1}f_{2}}{r_{1}f_{1}^{2}+2f_{1}f_{2}+r_{s}f_{2}^{2}}$$

where the subscripts refer to a specific monomer (i.e., monomer 1 and monomer 2) in the formulation.

## 3. Raman Measurements in Photopolymer Systems

### 3.1. Raman Microscopy

Raman microscopy provides options to gather information in multiple dimensions for photopolymer systems. However, to the author’s best knowledge, there is no microscopic system at this time with which real-time photopolymerization monitoring can be accomplished. One reason is that, to achieve high S/N, the exposure times are typically much higher for Raman microscopy than for fiber-optic-based Raman spectroscopy since laser power dramatically drops through the optical assembly of the microscope. For example, real-time Raman monitoring of METHB photopolymerizations used 0.5 s exposures with a Raman probehead delivering ~200 mW at the sample surface from a 785 nm laser, compared to 120 s exposures with a Raman confocal microscope delivering ~9 mW from the same laser [13]. In addition, it is difficult to route the illumination source to samples under the microscope. The distance between the sample and the microscope objectives is small, especially for the stronger magnifications, making it challenging to illuminate from the side. The illumination spot size needs to be much greater than the Raman laser spot size to avoid variations due to diffusion, eliminating routing the initiating beam through the objective itself. Illuminating from the bottom of the sample is also problematic since a notch filter for the wavelengths of the illumination source would need to be placed before the spectrometer to prevent those photons from reaching the detector and obscuring the Raman signal (or burning out the detector). Beer’s Law adds another confounding factor since the absorption profile is exponential with respect to distance, leaving little or no photons available for initiation at the top of the sample (i.e., the air/sample interface) where monitoring may take place. Thus, data collection is limited to discrete points, line scans, area maps, and depth profiles after illumination.

**Figure 4:**
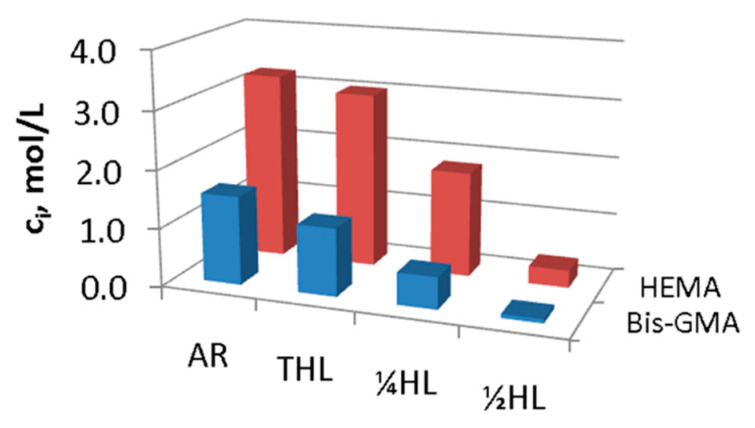
Absolute concentration (C_i_) of adhesive resin (AR) components from line scans of photopolymerized tooth fillings using Raman microscopy (adapted from Ref. [15]). HEMA predominantly comprises the polymer throughout the hybrid layer (HL), but the photopolymer penetration drops precipitously from the top (THL) to the halfway point (½HL) of the HL.

Despite the limitations in gathering real-time data, both conversion and composition information collected from Raman microscopy has proven fruitful in photopolymer studies. For example, with conversion calculations from depth profiles, the oxygen inhibition layer has been measured in epoxide–acrylate hybrid systems [13], in acrylate systems with LED sources [37], and in acrylate systems undergoing photopolymerization paired with electron-beam polymerization [38]. In dental resin systems, in situ conversion and relative composition data have been collected via line scans across the hybrid layer (i.e., where the adhesive resin penetrates the dentin of the tooth) for photopolymerized epoxides [39] and methacrylates [40]. In addition, a method to determine the absolute concentration of acrylate comonomers in the hybrid layer was developed, providing a clearer understanding of photopolymer infiltration in the tooth during line scans of tooth fillings (see Figure 4) [15]. Area mapping paired with depth profiling is particularly powerful when characterizing photopolymerized optical patterns. For example, area mapping has been used to gather composition and conversion data in nonlinear optical patterns formed in photopolymers [41] and to determine the density distribution of nanoparticles in photopolymer holographic gratings [42]. Furthermore, resolution advances in confocal Raman microscopy, such as super-resolution image restoration [43] and differential correlation [44], provide the opportunity to probe photopolymer composition and patterning at the nanoscale.

### 3.2. Physical Property Correlations

Because Raman spectroscopy is a non-destructive technique, there is the opportunity to relate spectral data to the resulting polymer physical properties by pairing with other analytical techniques. For example, photopolymerized thin films can be characterized first through Raman spectroscopy or microscopy and then through dynamic mechanical analysis (DMA) using a film tension clamp. Conversion and polymer structure can be correlated with important polymer properties such as glass transition temperature, crosslink density, and damping [26]. Simultaneous measurements can also be achieved. For example, Raman atomic force microscopes are available commercially and have been successfully applied to various polymer systems [45,46]. Rheometers can also be modified to measure viscosity and polymer concentration, as well as to identify the gel point, in polymerizing [47] and photopolymerizing [48] systems. Commercial rheometer–Raman systems are available with modifications to allow simultaneous illumination for photopolymerization.

## 4. Practical Tips

### 4.1. Experimental Considerations

**Figure 5:**
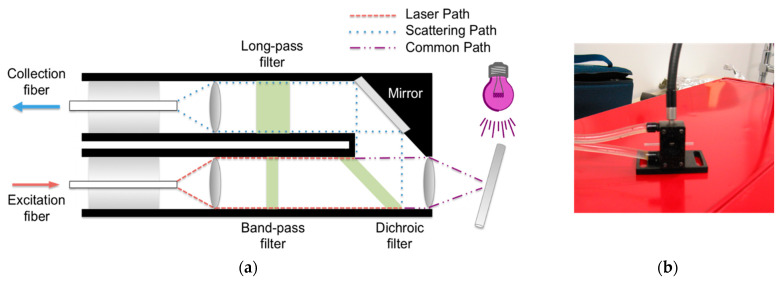
For a Raman system that collects 180° backscattered light (**a**), the initiating light source is placed perpendicular to the Raman laser beam (**b**) (adapted from Refs. [34,36]).

Developing an experimental protocol to collect Raman data requires thoughtful planning and some trial and error. Given the challenges of routing the illuminating light in a microscopy system (discussed above), handheld or fiber-optic-based Raman systems are needed to conduct real-time monitoring of photopolymerizations. With these systems, the illuminating light should be placed perpendicular to the Raman laser to minimize photons associated with the light source from reaching the detector and obscuring the Raman signal (or burning out the detector) (see Figure 5 for a representative example). Again, the illumination spot size should be larger than the Raman laser spot size, and the effective irradiance should be uniform across the illumination spot size (e.g., a collimating lens can be used for larger sample areas [26]). In addition, because the Raman signal is relatively small, the spectral collection should be conducted in a darkened area (e.g., in a room without windows and/or with the lights turned off). Even photons from a nearby computer monitor can be picked up by the sensitive detectors in Raman systems. The number of accumulations and exposure time will be based on the desired S/N, optical components in the system (e.g., collection fiber size), laser wavelength and power, and/or reaction speed. Moreover, substrates and sample holders for the photopolymerizable materials should be carefully chosen. Fluorescence is often produced from glass, so high-quality quartz is best. Metals (e.g., aluminum) and silicon also make excellent substrates since the few peaks they present in the Raman spectrum are at the low end of the fingerprint region (e.g., silicon’s peak is ~520 cm^−1^), which will not interfere with the peaks of interest in the photopolymerization systems. 

**Figure 6:**
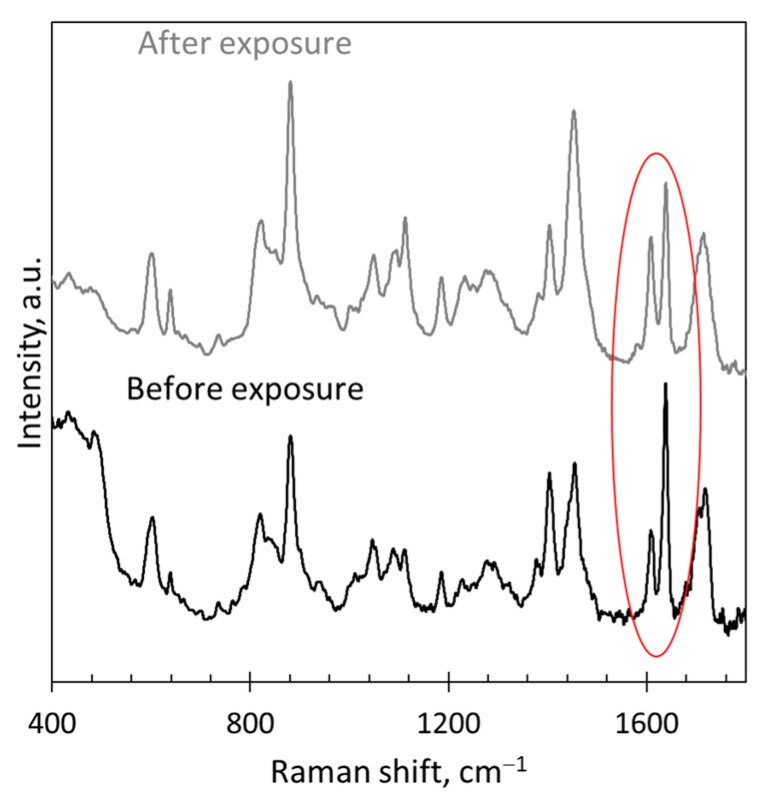
Photopolymerization is induced by 514 nm laser in Single Bond dental adhesive resin containing the co-initiator camphorquinone (adapted from Ref. [50]). Raman data were collected using a 785 nm laser before and after 60 s of exposure with the 514 nm laser. The reaction peak at 1640 cm^−1^ (circled) is noticeably decreased after exposure compared to the stable reference peak at 605 cm^−1^.

**Figure 7:**
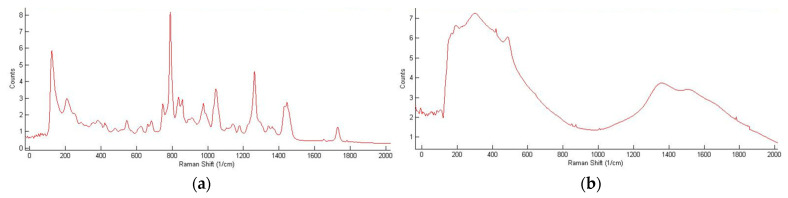
High temperatures can negatively impact the detail in a Raman spectrum. After photopolymerization of EEC, peaks in the Raman fingerprint region were well resolved (**a**); however, after annealing at 400 °C for 2.5 h, most Raman peaks disappeared (**b**) (adapted from Ref. [53]). The spectral resolution did not recover, even after 5 days at room temperature.

Moreover, careful consideration of the effect of the experimental setup and sample processing is required in order to avoid introducing unintended perturbations of the system that produce misleading spectral results. For example, photopolymerizable dental resins and composites include the co-initiator camphorquinone, which absorbs visible light from ~390 to ~520 nm [49]. This absorption spectrum overlaps slightly with the output from a 514 nm laser. If this laser is used to collect Raman data for these types of dental systems, the laser itself can artificially inflate the conversion measured by contributing to the photopolymerization. Under dark conditions (i.e., without an illumination source), 1 min of exposure to a 514 nm laser with a power output of ~0.33 mW at the sample surface resulted in over 20% of conversion of Single Bond dental adhesive resin (see Figure 6) [50]. In addition, tooth samples of photopolymerized dental resins and composites are often stored in aqueous solutions to mimic in vivo conditions and to keep the dentin hydrated. However, unpolymerized monomer can leach into the solution, resulting in a much larger (apparent) conversion of the remaining polymer material, which can be misinterpreted as dark polymerization [18]. When extending the Raman technique to acrylates undergoing electron-beam polymerization, the reference peak at 1070 cm^−1^ that worked well for photopolymerization of TMPTA was shown to be altered by the accelerated electrons [22]. Thus, a new reference peak that was stable under the electron beam had to be identified, and the addition of a comonomer with a phenyl ring enabled the use of Raman spectroscopy for this type of radiation curing as well. High temperatures may also disrupt acquisition of meaningful Raman spectra. If conducting an experiment at high temperatures, strong thermal emission may overpower the weaker Raman signal, requiring filtering of spectral data [51]. Even if a photopolymer sample is heated and then cooled before collecting data, a large background signal may develop (possibly due to microscopic surface roughening [52]), thereby obscuring sample peaks in the Raman spectrum. For example, Raman spectra of photopolymers after annealing or after multiple high-temperature DMA scans can remain void of any meaningful peaks even after leaving the samples at room temperature for several days (see Figure 7 for a representative example) [34].

### 4.2. Confocal Raman Microscopy

**Figure 8:**
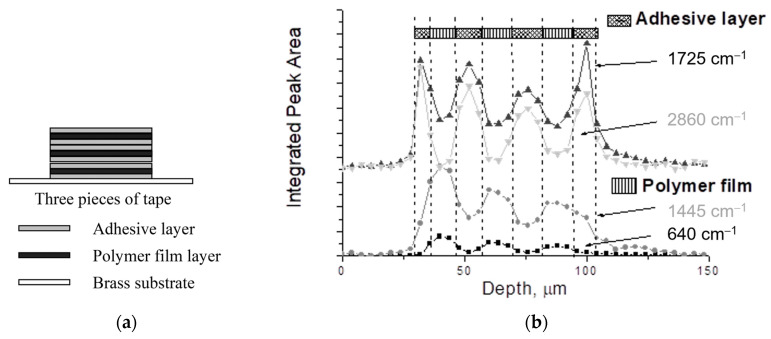
Several layers of double-sided tape (**a**) can provide good practice for perfecting the confocal Raman technique (**b**) (adapted from Ref. [36]).

Confocal Raman microscopy can be a challenging technique to perfect. Practicing with a known sample can be helpful in learning how to collect, analyze, and interpret data. For example, a stack of several layers of double-sided tape on a metal substrate (e.g., aluminum) is an excellent training tool [36]. The polymer film layers are characterized by Raman peaks at 640 and 1725 cm^−1^, and the adhesive layers are characterized by peaks at 1445 and 2860 cm^−1^ (see Figure 8). Depth resolution and spatial accuracy decrease with increasing depth because of differences between the refractive index of the sample and air. Thus, the depth the instrument reports may not be the actual depth in the sample. For example, the thickness of one piece of double-sided tape measured in the Raman training experiment (26 µm) is approximately half the real thickness measured using calipers (60 µm) (see Appendix A in Ref. [36]). If needed, mathematical models can be used to address the discrepancy [54,55,56]. Since the focal point of the Raman laser is not the same as the visual focal point of the microscope, it may be difficult to determine the air–surface interface when starting a depth profile. If the instrument software does not have an algorithm to find the interface, then a manual determination may be made by finding the depth at which the peak is maximum for a reference peak during a depth profile (see Appendix E in Ref. [57]). Because depth profiles can be time-consuming due to the high exposure times required for adequately high S/N, it is important to optimize the step size by ensuring that it is equal to or larger than the sampling resolution of the instrument. The full-width at half-maximum (FWHM) of a strong reference peak (e.g., 525 cm^−1^ from a silicon wafer) during a depth profile with 1 μm step sizes can provide the resolution of a particular experimental setup (e.g., collection fibers and microscope objective) used for the confocal measurement (see Appendix E in Ref. [57]).

## 5. Conclusions

Raman spectroscopy is a powerful tool for monitoring photopolymerizations and determining relationships among composition, processing, and polymer properties. As a spectroscopy-based technique, it provides both qualitative and quantitative information that is not possible with heat-based techniques such as photodifferential scanning calorimetry. Advances in detectors have made Raman spectroscopy competitive with its more ubiquitous sister technique, infrared spectroscopy, and it possesses its own advantages, such as ease of sample preparation, use in aqueous systems, and better resolution in microscopic applications.

When using Raman spectroscopy for online and/or real-time monitoring of photopolymerizations, the key concern is introducing the initiation light source in such a way as to avoid striking the Raman detector and to illuminate the volume of sample being investigated by the Raman laser. Careful selection of reference and reaction peaks is also paramount so that kinetic data, such as conversion and reaction rates, can be calculated with confidence. 

Although Raman confocal microscopy cannot be used to monitor photopolymerizations in real time, because of challenges in routing the initiation light source, the ability to perform point, line, and area scans, as well as depth profiles, can provide a wealth of information about the resulting photopolymers. Continued advances in microscope resolution and detector sensitivities, as well as signal processing, will allow further investigations on the sub-micron level, which are critical for applications such microfluidics and optical patterning. In addition, because Raman spectroscopy is a non-destructive technique, it can be used with other analytical techniques to develop structure–processing–property relationships that enable further applications of photopolymer systems. The availability of commercial systems pairing Raman with techniques such as with AFM and rheometry promise to provide exciting new insights for photopolymerization systems. 

The examples in this article are expected to serve as a springboard to inspire others to consider Raman spectroscopic techniques in their own photopolymer research and applications. The practical tips provided can expedite a successful implementation by avoiding the pitfalls and challenges that must be navigated for this special class of polymerizations. The new knowledge gained will facilitate increased use of photopolymerization and realization of its many benefits for industry and society alike.

## Figures and Tables

**Table 1:** Examples of reference and reaction peaks chosen from Raman spectra for common classes of monomers that are used in photopolymerization systems.

	Reference Peak		Reaction Peak		Representative Reference
	Peak (cm^−1^)	Moiety	Peak (cm^−1^)	Moiety	
trimethylolpropane triacrylate (TMPTA, acrylate)	1070	aliphatic groups	1636	vinyl C=C group	[22]
hydroxyethyl methacrylate (HEMA, methacrylate)	605	carbonyl (C–C–O) stretch	1640	vinyl C=C group	[18]
3,6,9,12-tetraoxatetradeca-1,13-diene (DVE-3, vinyl ether)	1458	wagging and bending of ethyl-ether hydrogens	1322	C–H bending of hydrogen attached to a vinyl carbon	[6]
3,4-epoxycyclohexylmethyl-3′,4′-epoxycyclohexanecarboxylate (EEC, cycloaliphatic epoxide)	1727	skeletal bending of the non-reactive CC=O group	789	asymmetric cycloaliphatic epoxide ring deformation	[23]
epoxidized polybutadiene oligomer (EPOH, aliphatic epoxide)	1640	1,2-vinyl C=C stretching vibrations	1271	in-phase breathing vibrations of oxirane ring	[23]
3-ethyl-3- [(2-ethylhexyloxy)methyl] oxetane (EHOX, oxetane)	1450	aliphatic groups	1150	oxetane ring	[24]

## Data Availability

The unpublished data presented in this review are available on request from the corresponding author.
